# Editorial: The attentional boost effect and related phenomena: new insights into the relation between attention and memory

**DOI:** 10.3389/fpsyg.2023.1226596

**Published:** 2023-06-06

**Authors:** Clelia Rossi-Arnaud, Daniele Saraulli, Pietro Spataro, Matthew Prull

**Affiliations:** ^1^Department of Psychology, Sapienza University, Rome, Italy; ^2^Department of Law, Economics, Politics and Modern Languages, Libera Università Maria Ss. Assunta, Rome, Italy; ^3^Department of Economy, Mercatorum University, Rome, Italy; ^4^Department of Psychology, Whitman College, Walla Walla, WA, United States

**Keywords:** attention, memory, cognition, attentional boost effect, recognition

The relation between attention and memory has been unanimously recognized as a key theme of modern cognitive psychology (e.g., Baddeley et al., 1984; Craik et al., 1996). Starting from this consideration, our Research Topic was aimed at putting together a selected set of papers which illustrate the latest advancements in the field. We were particularly interested in the examination of a phenomenon called the *Attentional Boost Effect* (ABE) but, as we will show below, the contributions extended to the discussion of a wide range of related findings.

The Research Topic includes a comprehensive review of the latest evidence on ABE and an updated version of the Dual-Task Interaction Model (Swallow et al.), previously proposed by Swallow and Jiang. Briefly, the ABE arises when stimuli encoded with to-be-responded items are remembered better than stimuli encoded with to-be-ignored items. This target-induced enhancement generalizes to both visual material (images: Swallow and Jiang, 2010; Sisk and Lee, 2022) and verbal material (Mulligan et al., 2014; Spataro et al., 2015). The Dual-Task Interaction model suggests that the detection of a target item leads to a transient increase in the amount of resources devoted to the perceptual processing of co-occurring stimuli, likely instantiated by the phasic release of norepinephrine from the locus coeruleus. The Dual-Task Interaction Model 2.0 maintains these tenets, but additionally proposes that the perceptual boost occurs whenever the state of the world does not coincide with the state of the neurocognitive system and a response is needed to bring them back into alignment. In addition, to account for emerging results showing that the ABE can extend to the encoding of contextual details (Turker and Swallow, 2019, 2022; Spataro et al., 2020, 2022; Mulligan et al., 2021), the model assumes that the target-related boost may enhance the formation of bound, multi-item representations in the MTL.

Consistent with the notion that subcortical noradrenergic structures play a key role in the ABE, the fMRI study by Moyal et al. found that auditory target detection produced broad physiological and neural effects. These include increases in phasic pupil responses, increases in the activation of the locus coeruleus and the ventral visual cortex, enhancements of the multi-voxel pattern classification of image category in the fusiform gyrus and parahippocampal gyrus, and enhancements in functional connectivity between the ventral visual cortex and the hippocampus. The latter result seems particularly relevant, because it suggests that the ABE-related manipulation benefits working memory maintenance and long-term memory encoding by enhancing communication between perceptual and medial temporal regions at behaviorally relevant times (such as when participants need to respond to target items).

An early finding in the ABE literature was that the memory enhancement following target detection was only significant for images that overlapped in time with the targets; no increase was observed when the images were presented 100 ms before or 100 ms after the targets (Swallow and Jiang, 2011). The study by Shimane et al. used a similar paradigm, in which participants responded to visual Go or No-go cues by pressing a key (the motor task) or by counting (the cognitive task). After each cue, two images were presented (a pre-item and a post-item). Memory for these images was later tested in a surprise recognition task. In line with the Swallow and Jiang (2011) conclusions, the results showed no significant difference in the recognition of pre- and post-items between Go and No-go trials in the motor task. However, in the cognitive task, post-items were better recognized in the Go than in the No-go trials. Moreover, No-go post-items were better memorized in the motor task than in the cognitive task. Jointly, these findings suggest that (a) in some conditions (i.e., with a non-motor task), target presentation may enhance memory for stimuli presented after its disappearance, and (b) covert motor engagement and response inhibition in No-go trials may promote memory encoding for task-irrelevant stimuli.

Another interesting finding in the ABE literature was that the memory enhancement induced by target detection may be reduced for distinctive stimuli that are already subject to heightened attention during an early encoding phase. Interactive effects of this type have been previously reported by Mulligan et al. (2014) for low-frequency words (but see Prull, 2019; for a different conclusion) and by Spataro et al. (2015) for words with rare orthographic features. An exception to this pattern has been reported in this Research Topic by LaPointe et al. They showed that perceptual degradation and target detection had significant, but independent effects on recognition memory, such that the ABE was similar in magnitude for clear and blurry words. Although these results are not necessarily inconsistent with the early-phase elevated attention hypothesis of the ABE, they nonetheless suggest that further research is needed to understand which manipulations are structurally redundant with the ABE.

As discussed above, the contributions included in this Research Topic cover a wide range of phenomena that are not limited to the ABE. Glicksohn et al., for example, demonstrated that the encoding of visual objects benefited from the association with unusual sounds, and that reactivating these sounds strengthened the entire multisensory representation, resulting in better memory for contextual details (such as the objects' locations). Muhmenthaler and Meier showed that objects presented during switch trials (i.e., trials in which participants had to switch between different classification tasks) were recognized worse than those presented during repeat trials, that the effect was still robust after a 1-week delay and that it was mainly due to recollection processes. Yu et al. used a three-phase sequential paradigm and found that the recognition of semantically-encoded words reduced the incidental encoding of new “foil” words, as compared to the recognition of orthographically-encoded words. They suggested that the detrimental effect occurred because semantic tasks relied primarily on recollection processes, whereas non-semantic tasks relied more strongly on familiarity processes. Lastly, a three-phase sequential paradigm was also adopted by Zhao et al., who reported that objects encoded with self-referential cues were recognized better than objects encoded with other-referential cues.

In conclusion, while we are still far from having a comprehensive understanding of the cognitive and neural underpinnings of the complex interactions between attention and memory, the studies briefly summarized in this editorial represent a promising starting point that should motivate enduring research efforts.

## Author contributions

PS wrote the first draft of the manuscript. All authors contributed to manuscript revision, read, and approved the submitted version.
